# A Strange Country

**Published:** 1882-11

**Authors:** 


### A STRANGE COUNTRY.

Visitors to Morocco find the country and its people marvelously strange and fresh. Theirs " are the rain and sunshine, and the ways of an old wisdom by our world forgot." Nowhere can a man be more conscious of the mere joy of living; nowhere be more impressed with the vanity and tedium of much that European civilization considers indispensable to the interests of life. From the hour you set foot in the country the present moment seems so full of delight that there is but the smallest desire to project life into anticipation. Time ceases to exist, or, at least, to be of any account.

It must be admitted, however, that the disposition of everybody in this lazy, lotos-eating land to act on this is at times a little troublesome. You have arranged to make an excursion to a town in the interior; you have fixed the day for starting—after having found that it must not be a Tuesday, for "*Tletsa felesta*" ("on the third day it fails"); nor on a Friday, for that is their Sabbath—you have been promised guides and mules by such a time; but do not expect them there, they will not come. You go to the man with whom you have made arrangements; he is probably doing nothing, and he has, probably, nothing to say in answer to your complaint, but "Please Allah, to-morrow." Yet, if you manage to set out on the third day, or before the week has run out, you will have been treated fairly well. They mean no neglect or disrespect; it is only their "Old World" way. Hence their proverb, "Once over the threshold the journey's half done."

For traveling there are, of course, neither carriages nor roads; the roads so called are only collocations of mule and camel tracks. Till within two or three years ago there was not a single wheeled vehicle in Morocco; but now there are known to be two—the one is a yellow, antediluvian gig, which the Sultan has procured somehow or other, and which figured sometime ago in a state procession, to the astonishment and delight of the spectators; the other is a wheelbarrow, which a gentleman recently took to Mogador, and of which the stalwart Moor to whom it was consigned so little surmised the use, that when he had filled it with mortar, according to instructions, he promptly shouldered it and strode off.

Traveling on muleback, however, in the large, well-padded saddles of the country, with stirrups like slippers, is no great hardship to either man or woman, and the way is constantly beguiled by some fresh illustration of Moorish life and manners—a company of noisy country-folk going to or coming from market; a troop of horsemen dashing past, with flowing white jelabs; the ghostly-looking silent cavalcade of an Arab sheik of the plains, and his harem, swathed from view, all but, perhaps, one lustrous eye; the astonished rustic girl, who hides her face, but not her legs; or the toilsome, almost naked peasant, dawdling along behind his aboriginal plow. A goat and a donkey may often be seen yoked together, and a woman has been seen in the place of a goat; but whatever the team, the plowing is always the same—a mere scoring of the soil, and yet it brings forth in abundance.—*Pall Mall Gazette.*

